# Multi-omics graph attention network reveals neuro-immune-tumor pathways in breast cancer liver depression syndrome

**DOI:** 10.3389/fimmu.2026.1873977

**Published:** 2026-09-22

**Authors:** Qianqian Guo, Jieting Chen, Geng Yang, Wanwei Jian, Xi Xiao, Zhenni Zhong, Yuqi Liang, Qian Zuo, Xiaojie Lin, Chunmin Yang, Yan Liang, Meilan Zhou, Liyu Chen, Rui Xu, Xuetao Wang, Yan Dai, Qianjun Chen

**Affiliations:** 1 State Key Laboratory of Traditional Chinese Medicine Syndrome/Breast Disease Specialist Hospital, The Second Affiliated Hospital of Guangzhou University of Chinese Medicine, Guangdong Provincial Hospital of Chinese Medicine, Guangdong Provincial Academy of Chinese Medical Sciences, Guangzhou, Guangdong, China; 2 Guangdong Provincial Key Laboratory of Clinical Research on Traditional Chinese Medicine Syndrome, Guangdong Provincial Hospital of Chinese Medicine, Guangzhou, Guangdong, China; 3 Postdoctoral Research Center, Guangdong Provincial Hospital of Chinese Medicine, Guangzhou, Guangdong, China; 4 Breast Disease Specialist Hospital, The Second Affiliated Hospital of Guangzhou University of Chinese Medicine, Guangdong Provincial Hospital of Chinese Medicine, Guangdong Provincial Academy of Chinese Medical Sciences, Guangzhou, Guangdong, China; 5 State Key Laboratory of Traditional Chinese Medicine Syndrome/Department of Radiation Therapy, The Second Affiliated Hospital of Guangzhou University of Chinese Medicine, Guangdong Provincial Hospital of Chinese Medicine, Guangdong Provincial Academy of Chinese Medical Sciences, Guangzhou, Guangdong, China; 6 Oncology Department, The Affiliated Traditional Chinese Medicine Hospital, Guangzhou Medical University, Guangzhou, Guangdong, China; 7 Guangdong Pharmaceutical University, Guangzhou, Guangdong, China; 8 The Second Clinical College, Guangzhou University of Chinese Medicine, Guangzhou, Guangdong, China; 9 Chinese Medicine Guangdong Laboratory, Guangzhou, Guangdong, China

**Keywords:** breast cancer, graph convolutional network, liver depression syndrome, MOGAT, multi-omics

## Abstract

**Background:**

Liver depression syndrome (LDS) is a core Chinese medicine (CM) syndrome implicated in the onset and progression of breast cancer, whose molecular mechanisms remain poorly understood, hindering further investigation.

**Methods:**

We prospectively enrolled 146 participants and conducted transcriptomic, proteomic, and metabolomic analyses using peripheral blood samples. A graph-attention-based multi-omics model embedded with a Transformer-derived multi-head self-attention module served as an exploratory tool to integrate multi-omics datasets. The identified candidate molecules were subsequently validated via enzyme-linked immunosorbent assay (ELISA).

**Results:**

In internal five-fold cross-validation, the Multi-Omics Graph Attention Network (MOGAT) achieved 96.6% accuracy in distinguishing LDS in breast cancer patients for biomarker-screening purposes, with proteomic features contributing predominantly to the classification. Moreover, both conventional multi-omics analyses and MOGAT analyses identified stimulator of interferon genes (STING), single immunoglobulin IL-1 receptor-related molecule (SIGIRR), tripartite motif-containing protein 56 (TRIM56), and anexelekto (AXL) as pivotal hub proteins, and subsequent ELISA validation confirmed differential expression of TRIM56 and AXL across pairwise comparisons. Multi-omics analysis identified noradrenaline as a candidate metabolite associated with BC-LDS related molecular alterations. The pathways underlying LDS in breast cancer were predominantly implicated in cytokine-cytokine receptor interactions, neuroactive ligand-receptor interaction, immune response regulation, and steroid hormone biosynthesis. These findings suggest candidate molecular features and a potential “neuro-immune-tumor-related” pathways associated with LDS in breast cancer.

**Conclusion:**

This study examined the integration of multi-omics data with MOGAT to investigate the biological underpinnings of CM syndrome. These findings provide a preliminary paradigm that combines CM syndrome omics data with deep learning approaches, offering a hypothesis-generating framework for deciphering the scientific mechanisms of CM syndrome.

**Clinical Trial Registration:**

http://itmctr.ccebtcm.org.cn/, identifier ITMCTR2025000446.

## 1 Introduction

Breast cancer is the most common malignancy and a leading cause of cancer-related mortality among women (1, 2). Previous studies have linked depression to breast cancer recurrence, all-cause mortality, and cancer-specific mortality (3, 4), and identified a high prevalence of anxiety and depression among Chinese breast cancer patients (5). Chinese medicine (CM) has emerged as an important complementary therapy for breast cancer patients (6, 7), proposing that the disease is influenced by emotional disturbance, improper diet, and insufficient innate endowment. Our previous research, including a systematic review (8), a multicenter clinical study (9), and a Delphi study (10), indicated that “Liver depression syndrome (characterized by emotional depression, frequent sighing, chest and lower abdominal distension or pain, breast distension or pain in women, and irregular menstruation)” (11) is the most common preoperative syndrome in breast cancer. However, the ambiguous scientific basis of this syndrome hampers its interpretation within modern medical frameworks, thereby limiting the understanding of the scientific connotations of CM.

In the era of precision medicine, the development of multi-omics-based biomarkers is essential for achieving its full potential (12, 13). Multi-omics data analysis is also critical in cancer molecular biology research (14). Scholars have proposed concepts such as precision CM syndrome medicine and CM phenomics (15, 16), which integrate traditional syndrome differentiation with the principles of precision medicine, including genomics, transcriptomics, proteomics, and metabolomics. This integration aims to achieve precise classification of CM syndromes and to clarify the biological basis of traditional CM theories. Traditional omics studies have primarily focused on single-omics analysis, overlooking cross-omics interactions and providing limited insight into multi-layer biological regulation (17, 18). Therefore, the limitations of single-omics analysis have driven the advancement of multi-omics technologies.

Multi-omics technologies have demonstrated considerable value and promising applications in CM research (19–21). For instance, transcriptomics, proteomics, and metabolomics have been applied to identify the “gene–protein–metabolite” network associated with phlegm and blood stasis syndrome in coronary heart disease (22). Although multi-omics approaches have significantly advanced syndrome biology, the complexity of multi-omics data necessitates further methodological optimization to strengthen the research framework of biology (23, 24). Current multi-omics integration strategies encompass statistical approaches, traditional machine learning, and deep learning methods (25). Leveraging machine learning, broadly including deep learning (26), multi-omics analysis facilitates biomarker identification and promotes precision medicine (27).

Deep learning employs neural networks to automatically learn feature representations, demonstrating robust performance in feature extraction and pattern recognition. It enables high accuracy and adaptability when integrating heterogeneous multi-omics data (28). A previous study proposed the MOGONET algorithm, an integrated omics analysis method based on graph convolutional neural networks, which supports omics-specific learning and cross-omics association learning to improve classification and interpretability (29). Furthermore, the emergence of Transformers and large-scale modeling techniques, characterized by attention mechanisms and enhanced learning capacity, has substantially advanced multi-omics integrative analysis (30). Wang et al. introduced a novel multi-omics integration method, MOSEGCN, which combines Transformer multi-head self-attention with graph convolutional networks to improve complex disease classification accuracy (31). Recent frameworks such as IGCN (32), further highlight the advantages of graph-based integration, cross-omics crosstalk and patient-level interpretability. These works establish methodological support for graph- and attention-oriented integration within prospective multi-omics clinical cohorts. However, most of these models were trained on public databases rather than on prospective clinical data.

With the continuing progress of deep learning in multi-omics research, we have explored its applications in multiple contexts. This study employs a graph convolutional network (GCN) and the Transformer framework to examine the interconnections among multi-omics data related to Liver depression syndrome (LDS) in breast cancer and candidate biological mechanisms associated with this syndrome. Using prospectively collected blood samples, this approach aimed to capture complex cross-omics interactions and heterogeneous data patterns, thereby advancing the application of modern biomedical research methods in the context of CM theory.

## 2 Materials and methods

### 2.1 Study design

Participants were recruited from Guangdong Provincial Hospital of Chinese Medicine (GPHCM). The inclusion criteria were: 1) patients met the diagnostic criteria for the disease; 2) female patients aged 18 to 70 years; and 3) voluntary participation with signed informed consent. Disease diagnoses were based on pathological findings. Syndrome diagnoses followed the Clinic Terminology of Traditional Chinese Medical Diagnosis and Treatment—Syndromes/Patterns, published by the State Bureau of Quality and Technical Supervision (33, 34), and also drew upon diagnostic criteria (35) established by our research team through systematic review (36), expert consensus, and clinical validation (**Supplementary Table S1**). Exclusion criteria were: 1) the concurrent presence of other malignant tumors; 2) the coexisting of severe primary diseases of the liver, kidney, hematopoietic system, etc.; 3) pregnancy or breastfeeding.

Patients were recruited from the Breast Departments of GPHCM. The study was approved by the Ethics Committee of GPHCM (approval no. BE2025-002-01). This study was conducted under the Helsinki Declaration and all participants provided written informed consent. Syndrome diagnoses were initially determined by the department chief and independently verified by two attending physicians. In cases of disagreement, an additional CM breast disease specialist with a deputy senior professional title or above was consulted. Participants were categorized into four groups based on disease and syndrome diagnoses: 1) breast cancer with Liver depression syndrome (BC-LDS) group; 2) breast cancer without Liver depression syndrome (BC-NLDS) group; 3) non-breast cancer with Liver depression syndrome (NBC-LDS) group; 4) non-breast cancer without Liver depression syndrome (NBC-NLDS) group. Data were recorded using an electronic case report form, and peripheral blood samples were collected after participants provided informed consent.

### 2.2 Multi-omics profiling and integrative analysis

Blood samples were collected in 5 ml EDTA-K2 anticoagulant tubes. For transcriptomic analysis, 3 ml of whole blood was used to isolate peripheral blood mononuclear cells (PBMCs) by the Ficoll method, and the remaining 2 ml was used for proteomic and untargeted metabolomic analyses. RNA extraction, library construction, and sequencing for transcriptomic, protein extraction and LC–MS/MS for proteomic, and metabolite extraction and subsequent LC–MS/MS detection for untargeted metabolomic were performed by Shanghai Majorbio Co., Ltd. Statistical analyses were conducted using R software (version 3.6.1) with corresponding analytical packages. Data visualization was implemented via Cytoscape (version 3.9.1) and an online bioinformatics platform (https://www.bioinformatics.com.cn).

#### 2.2.1 Transcriptome analysis

Libraries were sequenced on the NovaSeq X Plus platform to generate raw reads. Low-quality reads were removed using fastQC and Skewer to obtain clean reads, which were mapped to the human genome (GRCh38) using HISAT2. Gene expression levels were quantified using StringTie. Differentially expressed genes (DEGs) across the four groups were identified using DESeq2, with thresholds of *p* < 0.05 and fold change > 2. Weighted Gene Co-expression Network Analysis (WGCNA) modular analysis was performed based on all genes. Hierarchical clustering and transcript-per-million (TPM)-based expression analyses were performed using the pheatmap package in R. Gene Ontology (GO) enrichment and Kyoto Encyclopedia of Genes and Genomes (KEGG) pathway enrichment analyses of DEGs were conducted using clusterProfiler. Protein-protein interaction (PPI) network analysis of genes of interest was performed using the STRING database (http://string-db.org/).

#### 2.2.2 Proteome analysis

Proteins identified from reverse or contaminant databases were removed. For each sample, reporter ion intensities corresponding to the same gene were aggregated. Intensities were normalized using the median value for each sample to correct for differences in sample loading. Relative abundance was calculated as the ratio of sample abundance to internal reference abundance, followed by log2 transformation. The Wilcoxon rank-sum test was used to identify proteins with significant differences between groups. Significantly upregulated proteins were defined by *p* < 0.05 and fold change > 2, whereas downregulated proteins were defined by *p* < 0.05 and fold change < 0.5. GO functional enrichment and PPI network analyses were also conducted.

#### 2.2.3 Untargeted metabolome analysis

The data were imported into XCMS software (Scripps Research Institute, La Jolla, USA) for peak picking, peak grouping, and ion feature extraction. Metabolites were identified via LC-MS/MS using an in-house database established with authentic standards. The unpaired Student’s t-test was used to assess statistical significance. Metabolites with a variable influence on projection > 1 and a *p* < 0.05 were considered significantly different. WGCNA modular analysis and KEGG enrichment analysis for each module was conducted. For differential metabolites, the Human Metabolome Database (HMDB) was utilized for annotation, and KEGG–annotated pathways were visualized.

#### 2.2.4 Multi-omics data analysis

Pearson correlation analysis was conducted to calculate correlation coefficients between metabolites and genes, and a correlation network was constructed using ggplot2. KEGG pathway annotation was performed for both transcriptomic and metabolomic datasets, and co-annotated pathways between the two omics layers were further screened, from which associated metabolites and genes were extracted. Subsequently, we analyzed the abundance profiles of metabolites and genes involved in co-annotated pathways to identify molecules with significant differences (*p* < 0.05). For transcriptomic–proteomic integration, differentially expressed genes (fold change > 1; *p* < 0.05) and proteins (fold change > 1; *p* < 0.05) were identified in the BC-LDS and NBC-NLDS groups, and a nine-quadrant analysis was employed to identify molecules with concordant regulatory patterns (concurrently upregulated or downregulated). A network diagram was constructed to visualize the interactions among these molecules. KEGG pathway enrichment analysis was performed using the DEGs and differentially expressed proteins (DEPs), with significantly enriched pathways (*p* < 0.05) visualized in a bubble plot.

Integrated metabolomic and proteomic analyses were performed to identify co-enriched pathways and protein–metabolite interactions using Spearman correlation analysis. Subsequently, after filtering out low-abundance or invalid data, pairwise correlation coefficients among the features from the three omics layers were calculated. The correlations between genes and proteins were visualized in a heatmap, while the mean correlation coefficients between metabolites and genes/proteins were used to determine edge widths in the network representation, enabling cross-omics correlation analysis. Box-plot analysis was performed to compare noradrenaline levels across four groups.

### 2.3 Multi-omics graph attention network architecture

#### 2.3.1 Data preprocessing and feature selection

The raw expression matrices of the multi-omics datasets were organized with molecular features as rows and patient samples as columns. Samples were classified into two diagnostic groups: BC-LDS and the control group (comprising BC-NLDS, NBC-LDS, and NBC-NLDS), forming a binary classification framework. This classification target was used to identify a composite BC-LDS associated molecular pattern. Missing values, which comprised less than 2% of all measurements, were imputed using zero substitution to preserve the inherent sparsity of high-throughput mass spectrometry and RNA sequencing data.

For the initial exploratory analysis, feature filtering removed non-informative variables: zero or near-zero variance features (coefficient of variation < 0.01) were first discarded. Subsequently, discriminative features were selected by variance-based filtering followed by an Analysis of Variance (ANOVA) F-test, to reduce dimensionality and computational complexity. Based on established multi-omics integration methods and associated parameters (29), the final curated datasets contained 1,000 transcriptomic features, 1,000 proteomic features, and 500 metabolomic features (2,500 features in total). The t-distributed stochastic neighbor embedding (t-SNE) algorithm was used to visualize the inherent structure of the multi-omics features in a two-dimensional space. To address potential information leakage from supervised feature selection, we further performed leakage-free repeated nested cross-validation using the original full omics matrices. Within each training fold, missing values were replaced with zero, and the non-constant feature mask, ANOVA F-test selector retaining 1,000 transcriptomic, 1,000 proteomic, and 500 metabolomic features, and Min–Max scaling parameters were derived exclusively from the training data. The corresponding held-out fold was transformed using these training-derived procedures and parameters without refitting.

#### 2.3.2 Multi-omics graph attention network architecture

We developed a Multi-Omics Graph Attention Network (MOGAT) that integrates heterogeneous transcriptomic, proteomic, and metabolomic data through a hierarchical deep learning architecture inspired by recent advances in geometric deep learning and attention mechanisms (37, 38). The model addresses two fundamental challenges in multi-omics integration: 1) the heterogeneity of feature dimensions and statistical distributions across omics layers; 2) the need to capture complex interlayer dependencies while maintaining interpretability. The architecture adopts a hierarchical strategy in which omics-specific graph convolutional encoders first extract layer-specific representations. A multi-head attention mechanism subsequently learns adaptive cross-omics integration patterns, and a gating network dynamically assigns weights to each omics layer. Instead of constructing a newly designed attention module, MOGAT incorporated omics-specific GCN encoding, Transformer-based multi-head cross-attention and learnable gating weights for multi-omics datasets derived from this prospective CM syndrome cohort.

Each omics layer (transcriptome, proteome, and metabolome) was processed through an independent three-layer GCN encoder. For graph construction, patient samples were used as the computational units, and no external biological or patient-similarity graph was introduced. When no predefined adjacency matrix was supplied, an identity matrix with self-loops was used. Therefore, the encoder did not perform message passing between different patients and was interpreted as an omics-specific neural representation encoder in this study. A Leaky Rectified Linear Unit (Leaky ReLU) activation was applied to introduce non-linearity while mitigating gradient vanishing. Despite differing input dimensions, all three encoders projected their inputs into a unified 128-dimensional embedding space. The multi-head attention mechanism learned integration weights across layers, followed by a gating network that generated omics-level fusion weights. A fully connected layer was then used for representation refinement, along with layer normalization and dropout to prevent overfitting. Model hyperparameters were fixed before final internal cross-validation analysis.

#### 2.3.3 Model training and evaluation protocol

Stratified five-fold cross-validation was used for internal model evaluation, with approximately 80% of samples used for training and 20% reserved for testing in each fold. The modeling workflow and hyperparameters were fixed before the final cross-validation analysis. To address class imbalance (control group to BC-LDS ratio = 2.56:1), higher loss weights were assigned to the BC-LDS group using computed class weights. Model parameters were optimized using the Adam optimizer (learning rate 0.001, L_2_ regularization of 0.0001). Each fold’s weights were initialized using Xavier normal initialization and trained for 100 epochs in full-batch mode. All experiments were conducted on an NVIDIA RTX 3090 GPU using PyTorch 2.4.1. In the absence of an independent external validation cohort, model performance was reported as an internal exploratory estimate for reference in biomarker screening.

#### 2.3.4 Interpretability and feature importance analysis

To identify molecular features associated with MOGAT classification and generate candidate BC-LDS biomarkers, Gradient SHapley Additive exPlanations (SHAP) was applied as a *post hoc* interpretability method. Gradients were evaluated along interpolated paths between background and input values, using 200 Gradient SHAP sampling paths to estimate each feature’s contribution. SHAP values were calculated for each omics layer across the five-fold test sets, and global rankings were generated using mean absolute SHAP values to highlight candidate molecular features. SHAP and pathway analyses were performed after model evaluation and were used only for *post hoc* interpretation. Given the small sample size and high-dimensional omics data, SHAP-ranked features were interpreted as candidate markers requiring further validation. To assess robustness, we performed targeted sensitivity analyses by varying the number of selected features, missing-value imputation strategy, dropout, learning rate, weight decay, number of attention heads, representation depth, and random seed. SHAP feature stability was further evaluated using 1000 bootstrap resamples, and the frequency of each feature being included in the top 30 candidates was calculated.

#### 2.3.5 Performance evaluation and statistical analysis

Model performance was evaluated using five standard classification metrics: accuracy, precision, recall, F1-score, and the area under the curve of receiver operating characteristic (AUC-ROC). All metrics were calculated independently for each cross-validation fold and reported as mean ± standard deviation to assess model stability. To examine misclassification patterns, predictions from all five folds were aggregated to construct a comprehensive confusion matrix. The confusion matrix was normalized row-wise to visualize class-specific error rates, which is particularly important for imbalanced datasets in which overall accuracy may be misleading. Further, we calculated 95% confidence intervals (CIs) from the original five-fold cross-validation out-of-fold predictions using bootstrap resampling. Calibration performance was summarized using the Brier score and expected calibration error. We adopted a repeated nested cross validation framework for model evaluation. The outer layer applied stratified five-fold cross validation with three repetitions, and the inner layer implemented three-fold cross validation for hyperparameter optimization. Moreover, permutation tests based on the original full omics matrix were conducted with 100 label permutations. Each permutation strictly repeated the entire within-fold analysis pipeline, including data preprocessing, feature selection, model training and model testing.

To examine whether the internal discrimination reflected random label structure, we performed a label-shuffling sensitivity analysis. In 100 permutations, labels were randomly shuffled while the omics matrices were kept unchanged, and the same five-fold modelling workflow was repeated. AUC and balanced accuracy were compared against the corresponding null distributions to obtain empirical p values. To further examine the four-group design, supplementary factorial analyses were performed using the original preprocessed transcriptomic, proteomic, and metabolomic matrices. In addition, linear models assessed LDS-related molecular variations while adjusting for breast cancer status and its interaction with LDS status. P values were adjusted within each omics layer using the Benjamini-Hochberg false discovery rate (FDR) method. Details regarding network construction, training configurations and computational implementations were listed in **Supplementary Table S2**.

ANOVA scores in the predictive pipeline were used only for ranking features within each fold and were not interpreted as feature level statistical inference. For the supplementary differential and factorial analyses, Benjamini–Hochberg correction was applied separately within each omics layer and statistical comparison, with FDR < 0.05 considered significant. These analyses included a direct BC-LDS versus BC-NLDS comparison, an LDS model adjusted for breast cancer status, and an interaction model between cancer status and LDS. The MOGAT code has been uploaded to GitHub (https://github.com/younger1217/BRCA_LDS).

### 2.4 Enzyme-linked immunosorbent assay (ELISA) validation

Building upon the conventional multi-omics and MOGAT findings, we further narrowed our investigation to cytokine pathways and their hub proteins. Three pairwise comparisons were subsequently performed: BC-LDS versus NBC-NLDS, BC-LDS versus BC-NLDS, and NBC-LDS versus NBC-NLDS. Plasma concentrations of Stimulator of interferon genes 1 (STING1), Single immunoglobulin IL-1 receptor-related molecule (SIGIRR), Tripartite motif-containing protein 56 (TRIM56), and Anexelekto (AXL) were quantified using commercial sandwich ELISA kits (zhucaibio Co., Shanghai, China). Validation of RAB Guanine Nucleotide Exchange Factor 1 (RABGEF1) by ELISA was precluded by the lack of commercially available assay kits for this specific marker.

All assays were performed according to the manufacturer’s instructions. Absorbance was measured at 450 nm, and final concentrations were calculated from a standard curve. All samples were analyzed in triplicate. Pre-specified outlier management for ELISA data was outlined in the statistical analysis plan. Outliers in raw optical density values were identified using the Interquartile Range (IQR) method. Specifically, the first (Q1) and third quartiles (Q3) were calculated for each experimental group, and the IQR was defined as Q3–Q1. Data points falling below Q1–1.5×IQR or above Q3 + 1.5×IQR were considered outliers and excluded from the primary analysis. To ensure transparency and robustness, results were reported for both the full unprocessed dataset (raw data) and the dataset following IQR-based outlier removal. Furthermore, bootstrap analyses (10,000 resamples) were employed to estimate the 95% CIs for the between-group median differences.

## 3 Results

### 3.1 Clinical characteristics of the participants

The workflow of this study is shown in **Figure 1**, and the participants enrollment flowchart is presented in **Supplementary Figure S1**. A total of 146 participants were included in the omics analysis: 41 in the BC-LDS group, 31 in the BC-NLDS group, 40 in the NBC-LDS group, and 34 in the NBC-NLDS group. Clinical characteristics of all participants are summarized in **Supplementary Table S3**. Age analysis indicated that non-breast cancer participants were generally younger than breast cancer participants; consequently, most non-breast cancer participants were premenopausal. Baseline characteristics of the breast cancer participants are presented in **Supplementary Table S4**.

**Figure 1:**
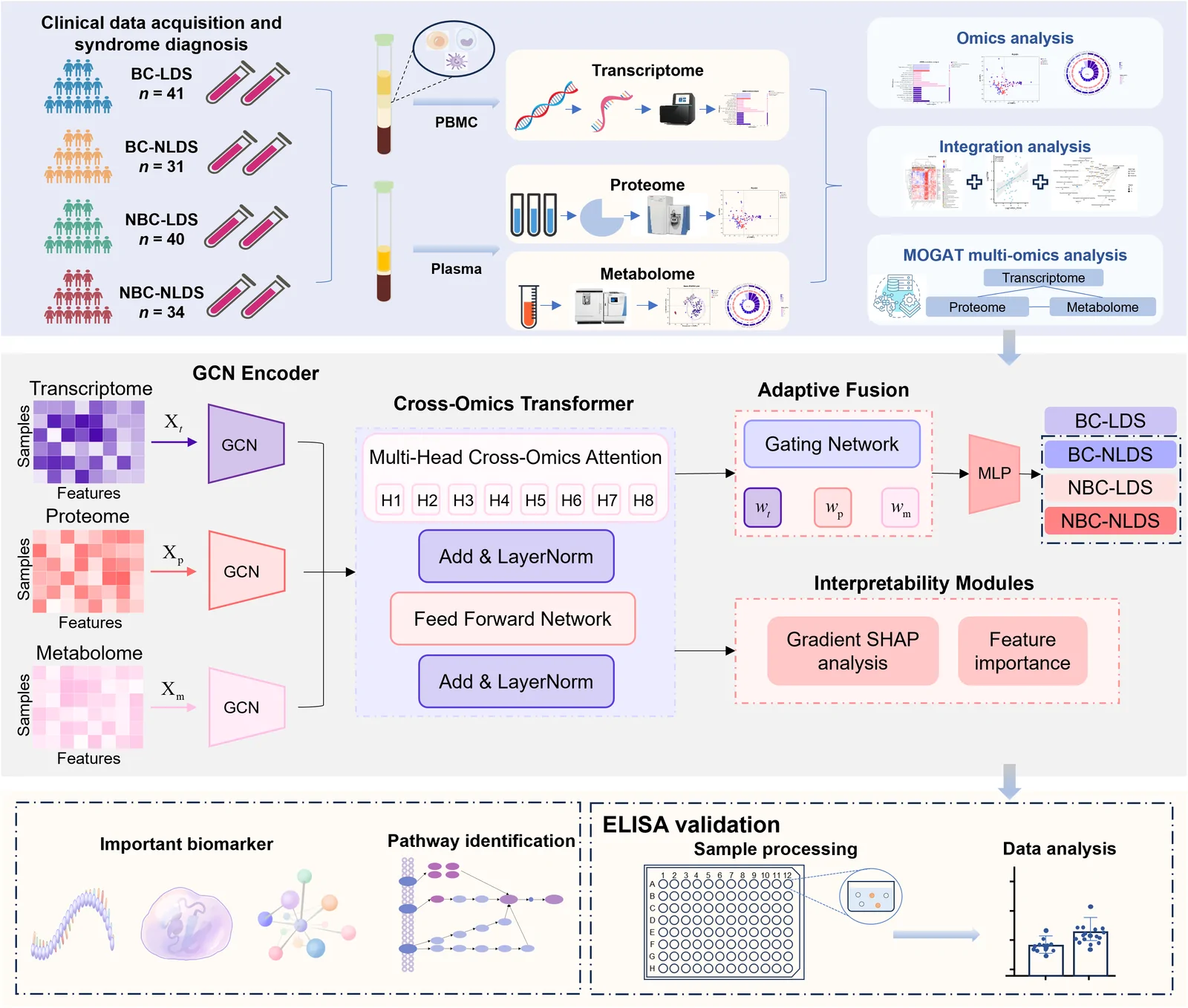
Schema flow chart of the study. BC, breast cancer; NBC, non-breast cancer; LDS, Liver depression syndrome; NLDS, non-Liver depression syndrome; PBMC, peripheral blood mononuclear cells; MOGAT, Multi-Omics Graph Attention Network; GCN, graph convolutional network; Add & LayerNorm, residual addition followed by layer normalization; MLP, multi-layer perceptron; SHAP, SHapley Additive exPlanations; ELISA, enzyme-linked immunosorbent assay.

### 3.2 Transcriptomic, proteomic, and metabolomic alterations characterizing BC-LDS

In the transcriptomic landscape, eight modules were delineated by WGCNA (**Figure 2A**). The red and brown modules exhibited the strongest positive and negative correlations with BC-LDS, respectively. After removing background effects, 263 BC-LDS genes were identified (**Figure 2B**). KEGG pathway enrichment analysis revealed significant enrichment in cytokine–cytokine receptor interaction and JAK–STAT signaling pathways (**Figure 2C**). PPI network analysis further identified JUN, IL10, and CAMP as key hubs (**Figure 2D**). The transcriptomic sequencing data generated in this study have been deposited in the NCBI SRA database under BioProject accession number PRJNA1497623.

**Figure 2:**
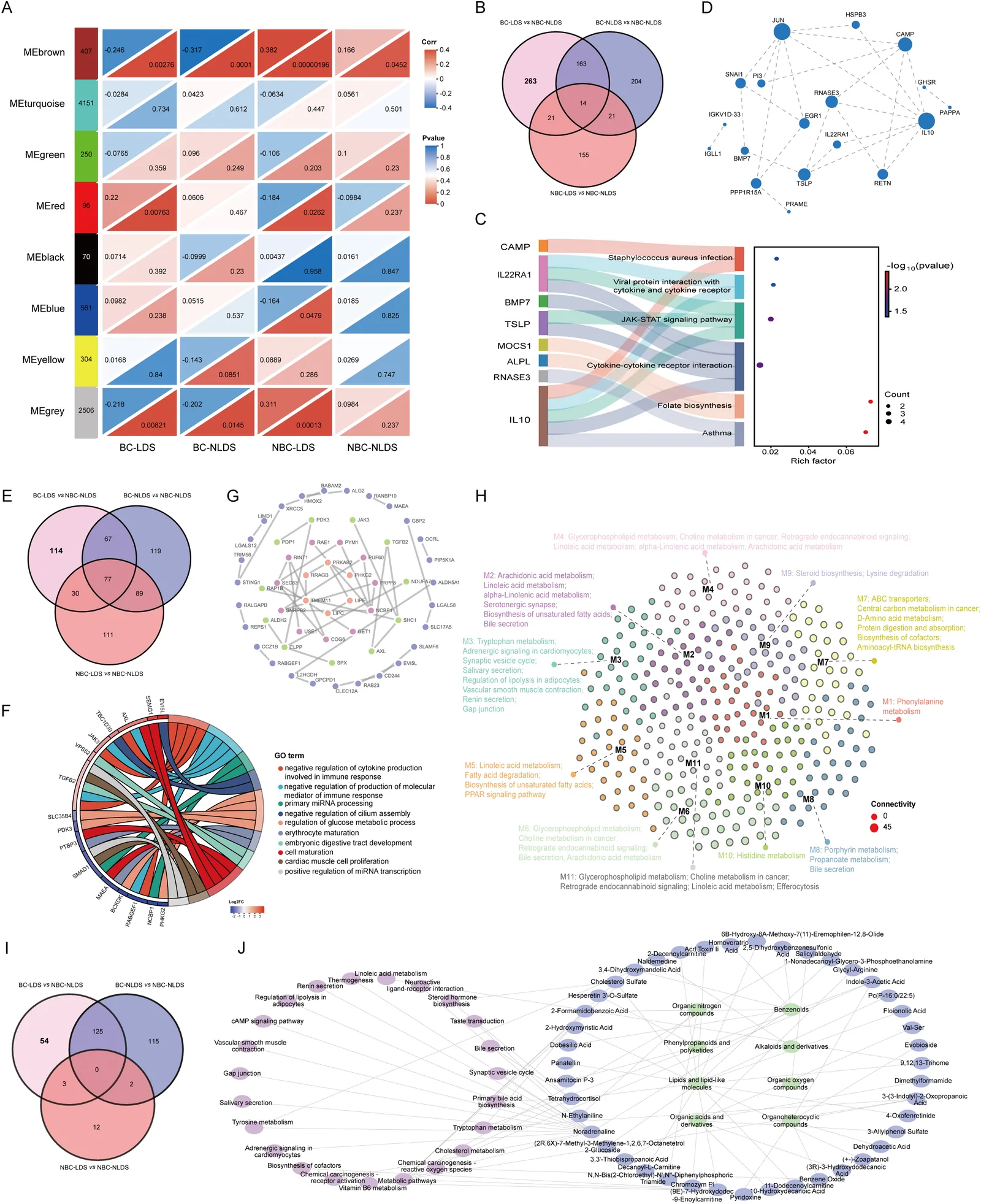
Features of breast cancer with Liver depression syndrome in transcriptomics, proteomics, and metabolomics. **(A)** Weighted gene co-expression network analysis (WGCNA) module analysis of the transcriptome. **(B)** Venn diagram of differentially expressed genes (DEGs) from all pairwise comparisons of the four groups, with the bolded number indicating the target gene set of BC-LDS. **(C)** KEGG enrichment analysis of BC-LDS target genes. **(D)** Protein-protein interaction (PPI) network analysis of BC-LDS target genes. **(E)** Venn diagram of differentially expressed proteins (DEPs) from all pairwise comparisons, with the bolded number indicating the target protein set of BC-LDS. **(F)** GO enrichment analysis of BC-LDS target proteins. **(G)** Network analysis of BC-LDS target proteins. **(H)** WGCNA module analysis of the metabolome and the KEGG pathways enriched in each module (top five pathways with *p* < 0.05). **(I)** Venn diagram of differentially abundant metabolites (DAMs) from all pairwise comparisons; the bold number indicates the BC-LDS target metabolite set. **(J)** Network of metabolites (blue), categories (green), and annotated KEGG pathways (purple) for the BC-LDS target metabolites. BC, breast cancer; NBC, non-breast cancer; LDS, Liver depression syndrome; NLDS, non-Liver depression syndrome.

Similarly, 114 characteristic proteins of BC-LDS were identified (**Figure 2E**). Enrichment analysis showed their involvement in immune-modulatory biological processes, particularly negative regulation of cytokine production (**Figure 2F**). The PPI network of these BC-LDS characteristic proteins is presented in **Figure 2G**. These proteins were broadly classified into lipid metabolism-related proteins (marked in orange) and proteins with more diverse biological functions.

For metabolomic profiling, WGCNA partitioned the data into 11 modules. The KEGG pathways enriched in these modules are depicted in **Figure 2H**, including choline metabolism in cancer. Subsequently, untargeted metabolomics identified 54 characteristic metabolites of BC-LDS (**Figure 2I**). Among them, 43 metabolites were annotated by the HMDB and classified into eight compound categories; notably, noradrenaline was implicated in the greatest number of annotated pathways (**Figure 2J**). Additional details of the single-omics analyses are provided in **Supplementary Figure S2**.

### 3.3 Integrative multi-omics network analysis reveals cross-layer regulatory mechanisms

Results of the pairwise comparative analyses are summarized in **Figure 3**. For the 54 metabolites and 263 genes identified in the preliminary single-omics analysis, Pearson correlation analysis revealed the top 10 metabolite-gene pairs with the strongest correlations, with the corresponding metabolites including Val-Ser and noradrenaline (**Figure 3A**). Transcriptomic and metabolomic comparisons identified 58 and 23 annotated pathways, respectively, and the five co-annotated pathways are presented in **Figure 3B**, comprising steroid hormone biosynthesis and neuroactive ligand–receptor interaction. We performed differential analysis of metabolites and genes involved in the five pathways between BC-LDS and NBC-NLDS. Among these, the metabolite noradrenaline and the gene CAMP showed statistically significant differential abundance and expression between the BC-LDS and NBC-NLDS groups, respectively (**Figure 3C**), suggesting their potential involvement in BC-LDS associated molecular alternations.

**Figure 3:**
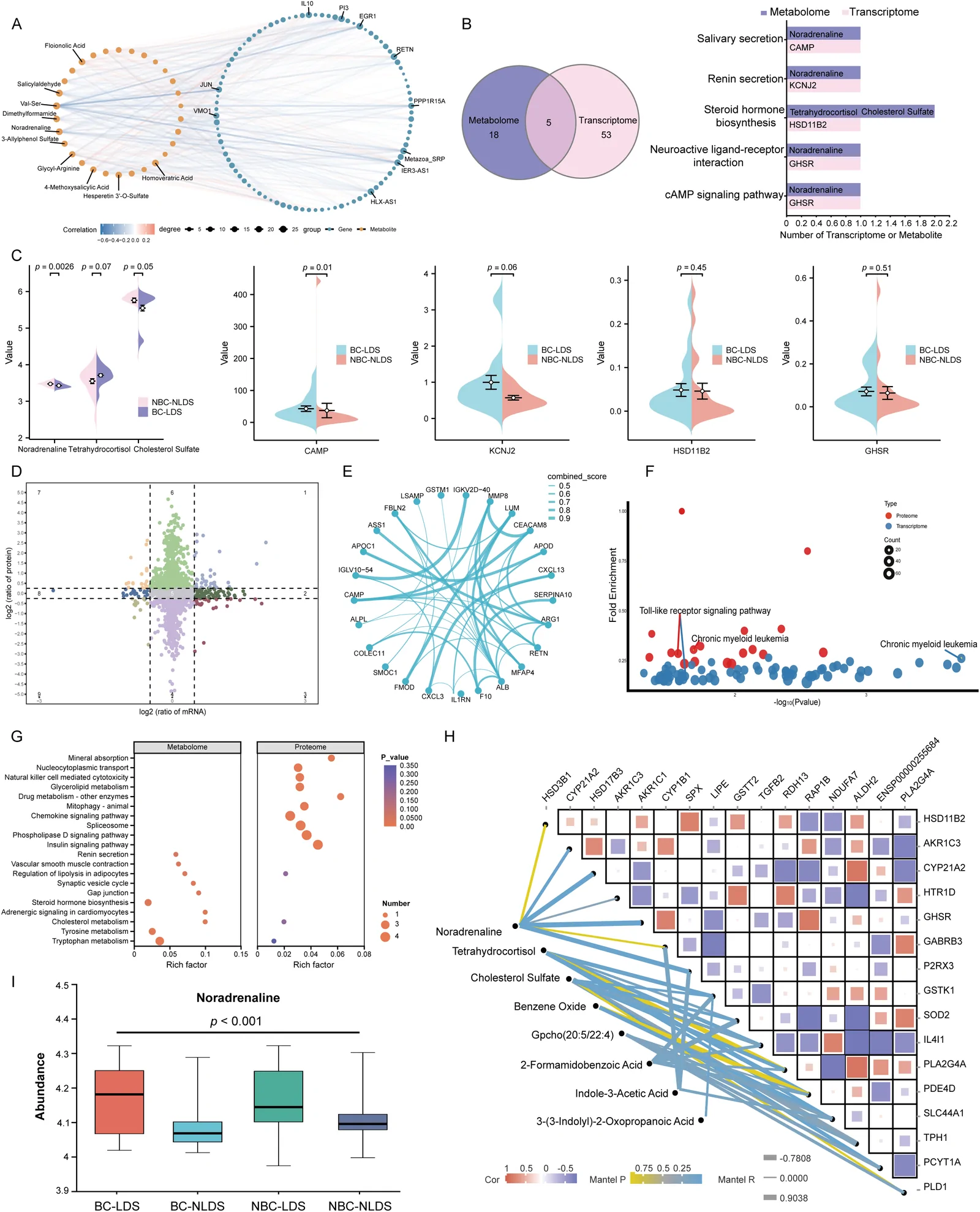
Integrated analysis of transcriptomic, proteomic, and metabolomic features of Liver depression syndrome in breast cancer. **(A)** Pearson correlation analysis of significant genes and metabolites (*p* < 0.05). The top 10 strongest correlations are labeled. **(B)** Co-annotated pathways of BC-LDS characteristic metabolites and genes. Genes and metabolites are labeled. **(C)** Differentially expressed metabolites and genes are shown within the five shared pathways between BC-LDS and NBC-NLDS. **(D)** Upregulation and downregulation of transcriptomic and proteomic features between BC-LDS and NBC-NLDS groups. **(E)** Correlation network of concurrently upregulated or downregulated genes and proteins. **(F)** KEGG enrichment analysis of differentially expressed genes and proteins between the BC-LDS and NBC-NLDS groups. **(G)** KEGG pathways co-enriched by BC-LDS characteristic metabolites and proteins. **(H)** Correlation analysis of BC-LDS characteristic metabolites with genes and proteins, with proteins on the horizontal axis and genes on the vertical axis. **(I)** Noradrenaline levels were compared among BC-LDS, BC-NLDS, NBC-LDS and NBC-NLDS groups. BC, breast cancer; NBC, non-breast cancer; LDS, Liver depression syndrome; NLDS, non-Liver depression syndrome.

To integrate the transcriptomic and proteomic profiles, a nine-quadrant analysis was performed to identify signatures with consistent regulatory patterns (**Figure 3D**). We specifically focused on genes and proteins exhibiting concordant expression—those concurrently upregulated (Quadrant 9) or downregulated (Quadrant 1) in the BC-LDS group relative to the NBC-NLDS group. A correlation network was subsequently constructed for these co-regulated molecules (**Figure 3E**). A total of 67 pathways from the transcriptomic analysis and 19 protein pathways were significantly enriched between the BC-LDS and NBC-NLDS groups (*p* < 0.05), with two pathways commonly enriched in both datasets: the Toll-like receptor signaling pathway and the chronic myeloid leukemia pathway (**Figure 3F**).

Based on the previously identified 114 proteins and 54 metabolites, the integrated analyses identified three co-enriched KEGG pathways: regulation of lipolysis in adipocytes, cholesterol metabolism, and tryptophan metabolism (**Figure 3G**). Through pairwise multi-omics integration analyses, we identified pathways closely associated with BC-LDS (**Supplementary Table S5**), as well as key molecules including metabolites such as noradrenaline, tetrahydrocortisol, and cholesterol sulfate. Correlation analysis across the three omics layers also revealed that noradrenaline was the metabolite that most strongly correlated with BC-LDS characteristic genes and proteins (**Figure 3H**), highlighting the potential relevance of neurotransmitter-related metabolites to BC-LDS molecular characteristics. Box-plot analysis demonstrated differences in noradrenaline abundance among the four groups, suggesting that noradrenaline may represent an LDS-associated metabolic feature rather than a breast-cancer-specific marker (**Figure 3I**).

### 3.4 Deep learning data results

The t-SNE algorithm effectively compressed high-dimensional features into a two-dimensional space (**Figure 4A**). In the model projection, the BC-LDS group (*n* = 41) and the control group (*n* = 105) exhibited clear separation in the embedding space. The 146 samples were divided into five folds while preserving the class ratio (28.1% BC-LDS, 71.9% control group). The model showed high exploratory performance in distinguishing the BC-LDS group from the control group across all evaluation metrics under five-fold cross-validation (**Figure 4B**). The aggregated confusion matrix for all 146 test predictions (**Figure 4C**) provided detailed insight into classification patterns. Among the 41 BC-LDS cases, 36 were correctly classified. All 105 control group participants were correctly classified (100% specificity, 0 false positives results), suggesting strong internal discriminatory ability between BC-LDS and the combined control group.

**Figure 4:**
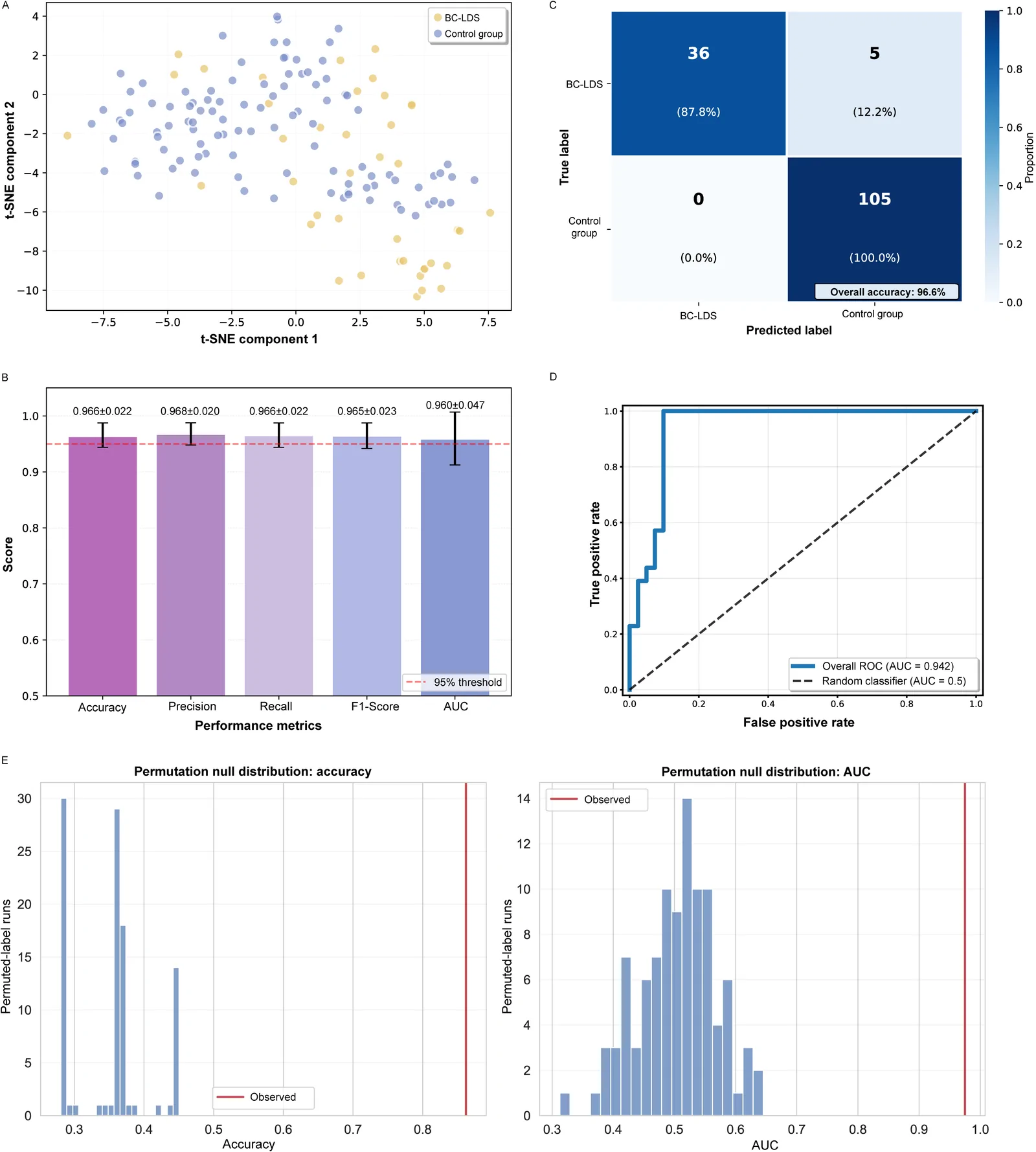
Multi-omics classification results of Liver depression syndrome in breast cancer. **(A)** t-SNE visualization of the multi-omics feature space. **(B)** Classification performance across five-fold cross-validation. Error bars represent the standard deviation across folds; the red dashed line marks the 95 percent performance threshold. **(C)** Aggregated confusion matrix across all test folds. **(D)** Overall ROC curve. **(E)** Histograms illustrate null distributions of accuracy (left) and AUC (right) across permuted label runs. t-SNE, t-distributed stochastic neighbor embedding; BC, breast cancer; LDS, Liver depression syndrome; ROC, receiver operating characteristic; AUC, area under the curve.

**Figure 4D** shows that the AUC of the model is 94.2% (95% CI: 0.878–0.988). Our model achieved an accuracy of 0.966 (95% CI: 0.932–0.993), a sensitivity of 0.878 (95% CI: 0.771–0.972), a specificity of 1.000 (95% CI: 1.000–1.000), and an F1-score of 0.935 (95% CI: 0.871–0.986). The Brier score and expected calibration error were 0.138 and 0.316, respectively. Label-shuffling sensitivity analysis was further performed to evaluate whether the observed classification performance could be attributed to random label permutation (**Figure 4E**). **Figure 4E** presents null distributions for accuracy and AUC from 100 permuted-label iterations. The mean null AUC reached 0.505, and the mean null balanced accuracy—calculated additionally to address class imbalance—was 0.501; both values were close to the chance level. The observed AUC and balanced accuracy were substantially higher than values derived from the corresponding null distributions, supporting the internal robustness of the classification results (empirical *p* = 0.0099 for both metrics).

To further rule out information leakage during feature selection, we reperformed model evaluation using the original full omics matrix. In the leakage free repeated nested cross validation, MOGAT yielded an AUC of 0.968, PR-AUC of 0.949, accuracy of 0.966, sensitivity of 0.927, specificity of 0.981, F1-score of 0.938, Brier score of 0.037, and ECE of 0.031 (**Supplementary Table S6**). In the raw matrix permutation test, the observed AUC and PR-AUC were 0.975 and 0.975, respectively, outperforming the corresponding null means of 0.503 and 0.304 (empirical p = 0.0099 for both; **Supplementary Table S7**).

As shown in **Figure 5A**, the proteomic representation received the largest gating-derived weight (47.9%) to the integrated model. Furthermore, the top 15 features ranked by mean absolute SHAP values were predominantly proteomic (including only one metabolite), suggesting that protein-level alterations may contribute substantially to distinguishing BC-LDS from the control group (**Figure 5B**). SHAP bootstrap analysis identified 65 features with a frequency of being included among the top-30 features ≥ 0.80, which were identified as SHAP-prioritized exploratory candidates in **Supplementary Table S8**. **Figure 5C** showed the top ten important features across the different omics types. Based on 2,500 selected features, GO and KEGG enrichment analyses were performed for the transcriptomic, proteomic, and metabolomic features, and the top ten pathways are shown in **Figure 5D**. Given the predominance of protein weights in the MOGAT matrix, we focused on the proteomic layer, identifying 16 shared pathways between conventional multi-omics analysis and the MOGAT workflow (**Figure 5E**). As shown in **Figure 5F**, these pathways were associated with cytokine signaling processes, leading to the identification of five candidate proteins: STING1, SIGIRR, TRIM56, RABGEF1, and AXL. The sensitivity analysis showed that the overall discriminative performance was retained under several perturbations, although performance was not invariant to all modelling choices (see **Supplementary Figure S3**).

**Figure 5:**
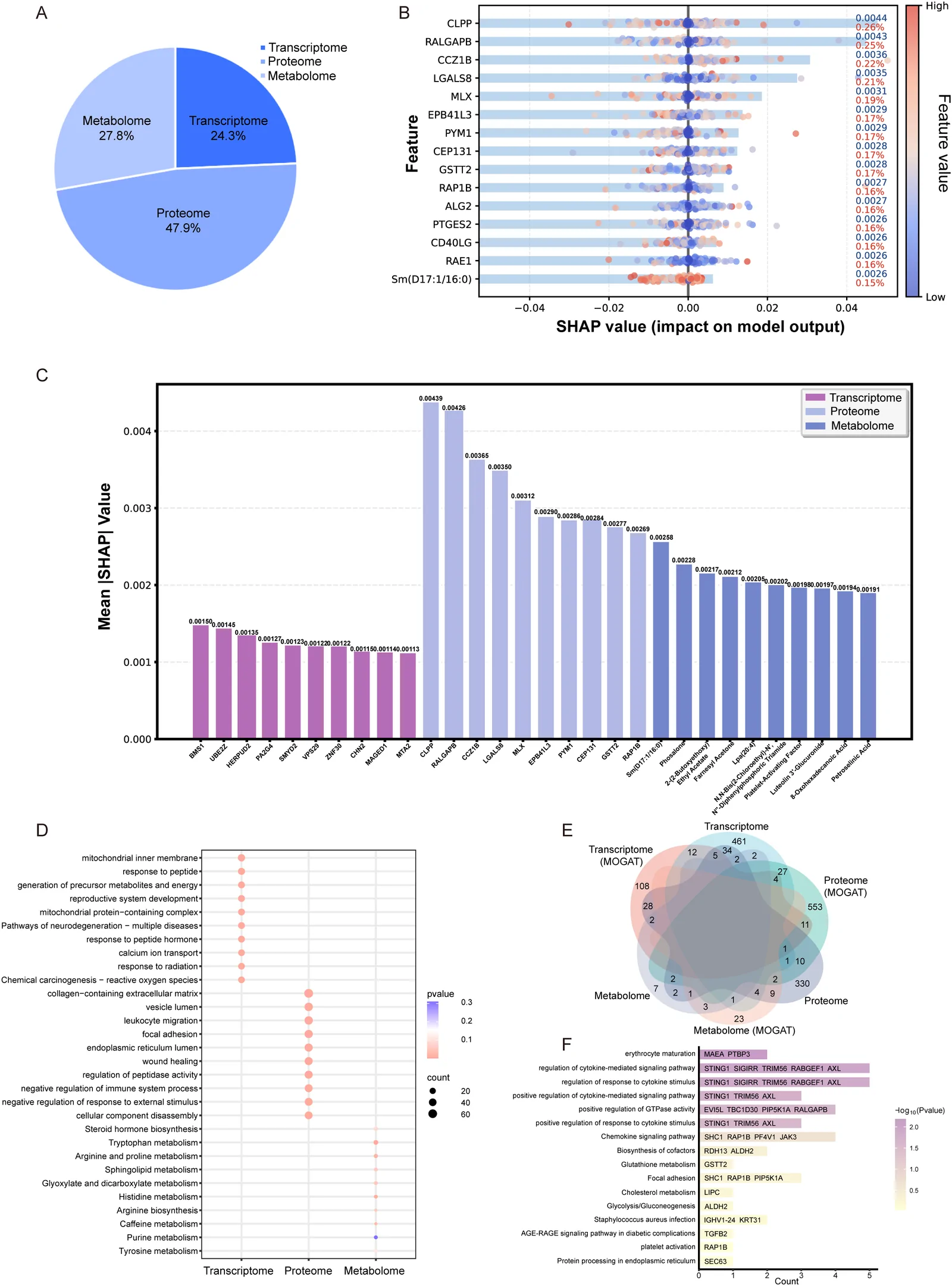
Exploratory omics candidates prioritized by MOGAT. **(A)** Gating-derived omics weights to the integrated model. **(B)** Top 15 features ranked by mean absolute SHAP value. SHAP values indicate each feature’s contribution to model predictions. The beeswarm distribution shows that, for a given feature, red points predominantly appearing on the right and blue points on the left indicate that high expression drives predictions toward BC-LDS, whereas the opposite pattern indicates that high expression drives predictions toward the control group. **(C)** Top ten most important features across different omics types. **(D)** Top ten GO and KEGG enriched pathways for the 2500 features derived from transcriptomic, proteomic, and metabolomic data. **(E)** Venn diagram showing the overlap between conventionally identified pathways and those identified by MOGAT. **(F)** Sixteen pathways shared between conventional proteomic analysis and MOGAT-based proteomic analysis. MOGAT, Multi-Omics Graph Attention Network; SHAP, SHapley Additive exPlanations.

Supplemental factorial analyses further supported the prominent role of the proteomic layer in the four-group design (see **Supplementary Table S9**). In the BC-LDS versus BC-NLDS comparison, 431 proteomic features remained significant at FDR < 0.05. In the LDS model adjusted for breast cancer status, 477 proteomic features remained significant at FDR < 0.05. An interaction model of breast-cancer status and LDS-status identified 569 proteomic features at FDR < 0.05. In contrast, features at the transcriptome and metabolome levels showed no statistical significance after FDR correction.

### 3.5 Validation by the enzyme-linked immunosorbent assay test

According to the conventional multi-omics and MOGAT analyses, the hub proteins associated with BC-LDS were identified. Plasma samples from four groups were subsequently used to validate protein expression. Statistical analysis revealed that, before and after outlier removal, significant differences between groups were observed in the levels of AXL and TRIM56 (**Figures 6A, B**). The results of the raw analyses, IQR-based analyses, and bootstrap analyses (see **Supplementary Table S10**) were highly consistent. Specifically, TRIM56 and AXL remained significantly different across pairwise groups comparisons, while statistically significant differences for STING1 and SIGIRR were observed in only a subset of these contrasts. Bootstrap-derived confidence intervals were consistent with these findings, supporting the robustness of the reported results.

**Figure 6:**
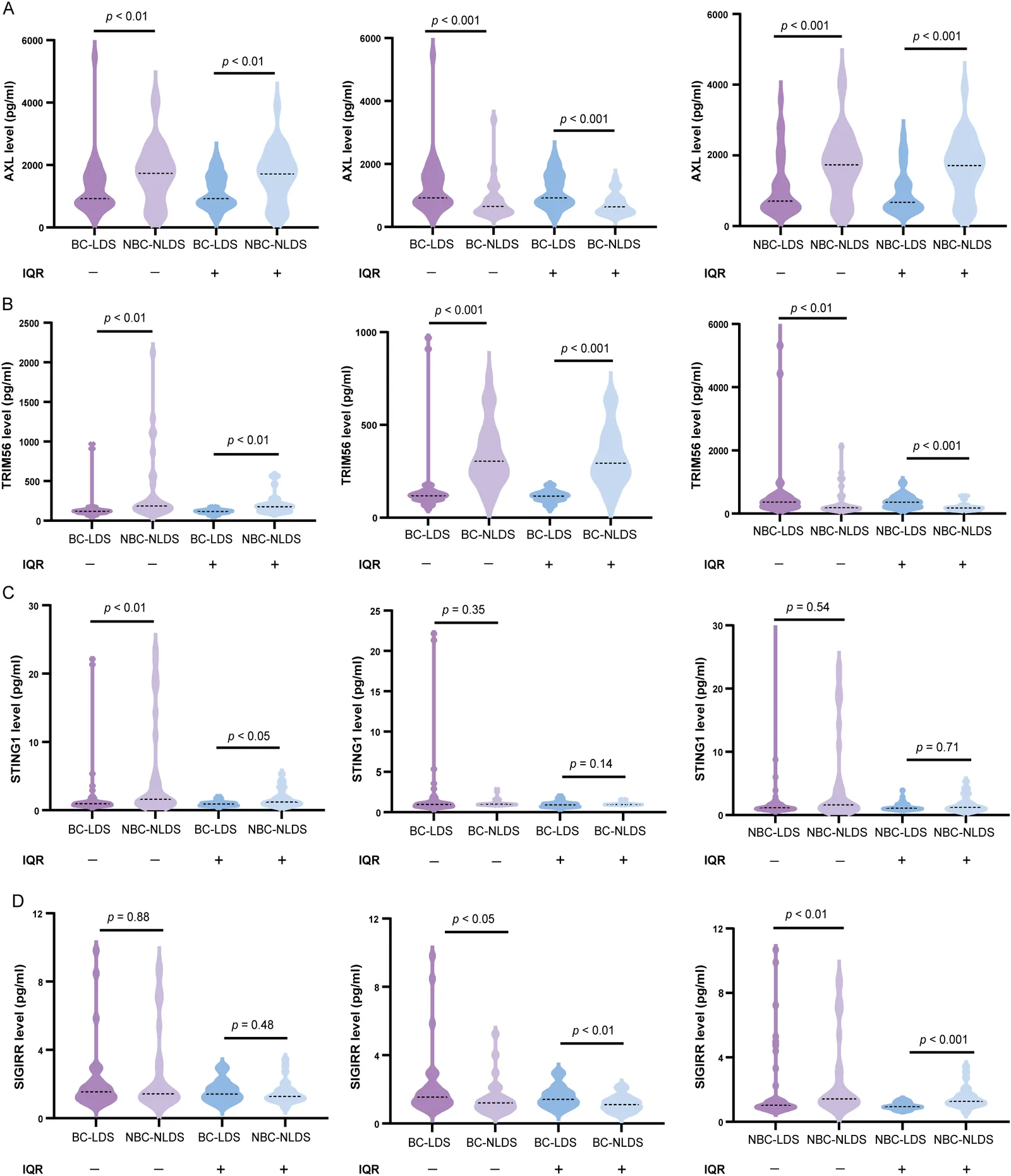
ELISA quantification validation. **(A–D)** ELISA measurements of AXL, TRIM56, STING1, and SIGIRR, with IQR-calculated upper outlier thresholds listed for each group below. AXL cutoffs: 2939.35 (BC-LDS), 1547.25 (BC-NLDS), 2581.85 (NBC-LDS), and 4098.14 (NBC-NLDS). TRIM56 cutoffs: 187.27 (BC-LDS), 732.22 (BC-NLDS), 1037.24 (NBC-LDS), and 623.95 (NBC-NLDS). STING1 cutoffs: 2.33 (BC-LDS), 1.57 (BC-NLDS), 3.25 (NBC-LDS), and 10.07 (NBC-NLDS). SIGIRR cutoffs: 3.42 (BC-LDS), 2.55 (BC-NLDS), 2.12 (NBC-LDS), and 5.36 (NBC-NLDS). Symbols: − denotes data before outlier exclusion, + denotes data after outlier exclusion; IQR, interquartile range; BC, breast cancer; NBC, non-breast cancer; LDS, Liver depression syndrome; NLDS, non-Liver depression syndrome.

## 4 Discussion

To our knowledge, our study performs an early implementation of deep learning in multi-omics integration to investigate CM syndromes, provides preliminary evidence for the “gene–protein–metabolite” network in LDS of breast cancer, and demonstrates the feasibility of exploring candidate mechanisms of such syndromes through an integrated approach. Moreover, this study provides a methodological example that links holistic CM principles with multi-omics technologies and deep learning to clarify the scientific connotation of CM syndromes. Beyond this, the study highlights the transformative potential of integrating CM with computational tools, offering data-driven insights for future personalized treatment research and candidate biomarker discovery, thereby advancing the modernization of CM within precision healthcare.

AXL is a receptor tyrosine kinase that activates downstream pathways including phosphatidylinositide 3-kinase/RAC-α serine/threonine protein kinase (PI3K/AKT) and p38 mitogen-activated protein kinase (MAPK) in breast cancer (39). A recent study indicated that nuclear AXL interacts with WRNIP1 (a DNA replication stress response factor) to mediate replication stress response, suggesting that targeting the AXL/WRNIP1 axis is a promising therapeutic strategy (40). Critically, AXL signaling is increasingly recognized for its role in shaping the immunosuppressive tumor microenvironment (41, 42) by modulating myeloid-derived suppressor cells and regulatory T cells, thereby facilitating immune evasion (43). In breast cancer, particularly triple-negative breast cancer, the STING serves as a central hub for immunomodulation (44). A promising therapeutic strategy would combine STING agonists with IL-6 inhibitors (45) or focus on the cyclic GMP-AMP synthase (cGAS)–STING signaling pathway (46) in cancer immunotherapy, providing novel directions for improving clinical outcomes. While the function of TRIM56 and SIGIRR in breast cancer remains poorly characterized, one study reported that TRIM56 acts as a tumor-promoting factor in estrogen receptor alpha (ERα)-positive breast cancer by enhancing ERα signaling and protein stability, which correlates with poor prognosis (47). Therefore, these findings prompt us to hypothesize that emotional stress associated with LDS may be linked to breast cancer related molecular alterations through neuroendocrine-immune-associated pathways, which warrants further experimental investigation.

The multi-omics analysis highlights the involvement of neurotransmitter-associated signaling in BC-LDS. Enrichment of targets related to cytokine interactions, neuroactive ligand-receptor interactions, and pathways related to immune response regulation collectively indicated potential “neuro–immune–tumor” associated molecular patterns, which guided our subsequent investigations. Neurons within the tumor microenvironment have considerable research significance, as they modulate immune responses by releasing neurotransmitters and neuropeptides that influence immune cell activity (48, 49). Additionally, neuronal components may affect tumor responses to therapies (such as chemotherapy, radiotherapy, and immunotherapy), and specific neuronal markers may serve as biomarkers for prognosis or therapeutic targeting (50).

Integrating diverse multi-omics data is essential for gaining comprehensive biological insights and understanding complex biological systems (51). In the context of LDS in breast cancer, multi-omics integration also serves as a key approach to decipher molecular mechanisms, and this study suggests that proteins carry substantial weight across several omics’ layers. Current studies also emphasize the potential of proteogenomic—which integrates next-generation DNA and RNA sequencing with mass spectrometry-based proteomics—in the clinical investigation of breast cancer, thereby providing a valuable reference for future multi-omics research on this disease (52). With rapid advancements in biotechnology, a variety of deep learning frameworks have been employed to integrate unstructured and structured omics data (53–55). In breast cancer, integrating multi-omics features from pretreatment breast tumor biopsies into machine learning models can predict treatment response (56), although such application remain rare in CM syndrome research.

With the advent of systems biology, omics technologies (including transcriptomics, proteomics, and metabolomics) combined with bioinformatics and computational tools have been widely and effectively applied to investigations of the biological basis of CM syndromes. These approaches provide valuable insights into the quantities and interactions of biomolecules underlying CM syndromes (57). For example, the biological basis of the CM syndromes Spleen *qi* deficiency and Spleen and Stomach damp-heat syndrome has been elucidated by integrating miRNA and mRNA analyses (58). Additionally, metabolomic and proteomic analyses of CM dampness syndrome (*Shi Zheng*) have also been reported (59). These studies not only enrich the theoretical understanding of the biological basis of CM syndromes but also emphasize the importance of multi-omics integration in CM research. Consequently, the development of advanced techniques for multi-omics integration has become a critical area requiring further exploration.

Recent graph and attention based multi-omics frameworks, including MOGONET (29), MOSEGCN (31), and IGCN (32), have advanced omics specific representation learning, cross-omics information flow, and interpretable multi-modal fusion. These studies provide an important methodological foundation for the present work. Rather than building a diagnostic classifier using MOGAT, we adopted a multi-omics integration strategy. We prioritized candidate molecules linked to BC-LDS by comparing participants with this composite phenotype against the rest of the cohort. In this context, our study extends graph and attention based multi-omics integration to prospectively collected CM syndrome data and links model attribution to candidate neuroendocrine-immune-tumor pathways in breast cancer. The main value of MOGAT in the present study lies in organizing heterogeneous transcriptomic, proteomic, and metabolomic signals and linking model attribution with candidate biological pathways relevant to LDS in breast cancer.

Building upon the identification of core molecular targets in breast cancer-associated LDS through multi-omics and graph neural network analyses, future research should validate these targets *in vivo*, *in vitro*, and in clinical cohorts to determine their mechanisms of action in tumor progression and within the tumor microenvironment. Relevant studies also provide methodological references for computational modeling and validation (60, 61). Moreover, delineating the relationship between CM syndrome symptoms and these molecular targets will deepen our understanding of the underlying mechanisms and facilitate the integration of CM with molecular medicine. In addition, multimodal data in molecular cell biology may be incorporated (62), such as digitalized CM diagnostic metrics combined with omics data, to construct diagnostic models for this syndrome and to develop intelligent prescriptions targeting key molecules through artificial intelligence–guided technologies. Future studies could also draw on prior research (63) to further investigate the action mechanisms of candidate targets by integrating organoids and deep learning technologies. This approach will support early intervention and promote clinical translation. Collectively, these strategies integrate the holistic philosophy of CM with molecular precision and advance personalized oncology therapeutics based on integrative medical paradigms.

In this study, we have computed bootstrap confidence intervals and calibration metrics to conduct initial internal assessment for our dataset. Rigorous quantification regarding main effects linked to breast cancer status, LDS status and their mutual interactions will be achieved with larger scale four group cohorts with balanced participants, covariate-adjusted factorial models, nested analyses and independent external cohorts in future investigations. Further efforts including repeated nested validation, external validation and comprehensive uncertainty evaluation across imputation, batch correction, ELISA detection and pathway enrichment will depend on well-powered cohorts equipped with complete technical metadata. Taken together, our results pave the way for subsequent external validation and comparative analyses by recruiting expanded and balanced participant cohorts in follow-up projects.

Several strengths and limitations of this study should be noted. In cohort studies, inconsistent omics data across individuals often reduces available samples and interferes with cross omics correlation analysis (64). The present study integrated transcriptomic, proteomic and metabolomic profiling from identical patient samples. This unified design reduces individual variation, minimizes confounding effects, and improves the biological authenticity and interpretability of multi-omics findings. Regarding limitations, the modest sample size, high-dimensional omics features, and lack of external validation may introduce optimistic bias in model performance. While the initial 2500 feature set stemmed from exploratory feature prioritization, newly added leakage free repeated nested cross validation and permutation tests on raw matrices confirm classification signals do not arise merely from supervised feature-selection leakage or random label effects. Built with an identity adjacency matrix containing only self-loops, the model does not capture patient-patient graph structure or graph-mediated biological interactions. All assessments remain confined to internal cohort validation, so independent external validation is necessary. The current sample supports multi-omics analysis, yet larger cohorts are essential for precision-medicine progress (65). Scarce public syndrome-biomarker databases further limit external model validation. To mitigate these limitations, we validated the omics results using ELISA. In addition, our research team previously developed an animal model for breast cancer with depressive symptoms and has applied for a patent (patent number: ZL202110391652.2). However, the animal model for BC-LDS remains under development and therefore was not used for validation in the present study.

## 5 Conclusions

In summary, this study applies multi-omics strategies alongside the MOGAT framework to examine molecular features of CM syndromes. Beyond identifying candidate important biomarkers and pivotal pathways linked to BC-LDS, our work builds a feasible research workflow that incorporates syndrome related omics datasets and deep learning algorithms. These findings also bring innovative methodological insights for elucidating the underlying mechanisms of CM syndromes.

## Data Availability

The datasets presented in this study can be found in online repositories. The names of the repository/repositories and accession number(s) can be found in the article/**Supplementary Material**.

## Glossary

ANOVA: Analysis of Variance
AUC: Area under the curve
AXL: Anexelekto
BC-LDS: Breast cancer with Liver depression syndrome
BC-NLDS: Breast cancer without Liver depression syndrome
CI: Confidence intervals
CM: Chinese medicine
DEGs: Differentially expressed genes
DEPs: Differentially expressed proteins
ELISA: Enzyme-linked immunosorbent assay
ERα: Estrogen receptor alpha
FDR: False discovery rate
GCN: Graph convolutional network
GO: Gene Ontology
GPHCM: Guangdong Provincial Hospital of Chinese Medicine
HMDB: Human Metabolome Database
IQR: Interquartile range
KEGG: Kyoto Encyclopedia of Genes and Genomes
LDS: Liver depression syndrome
Leaky ReLU: Leaky Rectified Linear Unit
MAPK: Mitogen-activated protein kinase
MOGAT: Multi-Omics Graph Attention Network
NBC-LDS: Non-breast cancer with Liver depression syndrome
NBC-NLDS: Non-breast cancer without Liver depression syndrome
PBMCs: Peripheral blood mononuclear cells
PI3K/AKT: Phosphatidylinositide 3-kinase/RAC-α serine/threonine protein kinase
PPI: Protein-protein interaction
RABGEF1: RAB Guanine Nucleotide Exchange Factor 1
ROC: Receiver operating characteristic
SHAP: SHapley Additive exPlanations
SIGIRR: Single immunoglobulin IL-1 receptor-related molecule
STING: Stimulator of interferon genes
TPM: Transcript-per-million
TRIM56: Tripartite motif-containing protein 56
t-SNE: t-distributed stochastic neighbor embedding
WGCNA: Weighted Gene Co-expression Network Analysis
