# Supplementary material for: Multi-omics graph attention network reveals neuro-immune-tumor pathways in breast cancer liver depression syndrome
Source: Front Immunol. 2026 Sep 22;17:1873977. doi: 10.3389/fimmu.2026.1873977 (PMC13638592; doi:10.3389/fimmu.2026.1873977)
Supplement: Supplementary file 1 [file DataSheet2.docx]

Table S1 The Liver depression syndrome diagnostic criteria.

| **Sources** | **Syndrome description** |
| --- | --- |
| Clinic Terminology of Traditional Chinese Medical Diagnosis and Treatment—Syndromes GB/T 16751.2-1997* | Liver depression (*qi* stagnation) syndrome: a condition in which the Liver fails to ensure free coursing, causing *qi* to stagnate. It is characterized by emotional depression, frequent sighing, distending pain and stuffiness in the chest, hypochondria or lower abdomen, premenstrual breast distending pain, irregular menstruation, and a stringy pulse. |
| Clinic Terminology of Traditional Chinese Medical Diagnosis and Treatment—Syndromes/patterns GB/T 16751.2-2021** | Liver depression syndrome: a general term for a group of patterns arising when emotional injury impairs the Liver’s function of free coursing, which may further transform into heat or fire scorching *yin*-blood, or may be complicated by damp-heat, blood stasis, or phlegm-turbidity. |
| Expert consensus on the Chinese medicine syndrome diagnosis of Liver depression syndrome in breast cancer*** | Primary signs and symptoms:   1. Impatience or frequent sighing; 2. Distending pain in the chest and hypochondria; 3. Symptoms vary with emotional states; 4. Stringy pulse;   Secondary signs and symptoms:   5. Breast mass, or hard breast mass, or the boundary of the mass is unclear; 6. Depression, or anger, or vexation, or dysphoria.   Diagnostic criteria: The syndrome is confirmed when any two of items 1–4 plus any one of items 5–6 are present. |

*The State Bureau of Quality and Technical Supervision. Clinic Terminology of Traditional Chinese Medical Diagnosis and Treatment-Syndromes. 1997.

**The State Bureau of Quality and Technical Supervision. Clinic Terminology of Traditional Chinese Medical Diagnosis and Treatment-Syndromes/patterns. 2021.

***This expert consensus was published in the following study: P. Wu, Q. Guo, X. Pei, L. Lou, W. Bian, H. Situ, H. Chen, L. Liu, X. Meng, R. Zhuo, L. Ren, C. Chen, Developing an expert consensus on the TCM diagnostic criteria for Liver depression syndrome in breast cancer using the Delphi method and analytic hierarchy process, Journal of Guangzhou University of Traditional Chinese Medicine. 43 (2026) 1399-1408.

Table S2 Model hyperparameters and experimental settings for multi-omics MOGAT.

| **Item** | **Value** | **Notes** |
| --- | --- | --- |
| Analysis role | Exploratory MOGAT classification | BC-LDS versus combined comparator; not a factorial LDS-specific inference |
| Omics layers | Transcriptome; proteomic; metabolomic | Input files: BRCA_LDS/1_tr.csv, 2_tr.csv, 3_tr.csv |
| Selected feature counts | 1000 transcriptomic; 1000 proteomic; 500 metabolomic | Total 2500 features |
| Cross-validation | Stratified five-fold | Fold-internal preprocessing described in Methods |
| Random seed | 42 | Used for reproducible splitting and attribution sampling where applicable |
| Hidden dimension | 128 | Three omics-specific encoder layers project to shared embedding size |
| Attention heads | 8 | Cross-omics multi-head attention |
| Dropout | 0.3 | Classifier uses an additional dropout multiplier in the first classifier block |
| Optimizer | Adam | Learning rate 0.001; L2 regularization 0.0001 |
| Epochs | 100 | Full-batch training |
| Class imbalance | Class weights | BC-LDS class weighted in loss function |
| Graph adjacency | Identity adjacency with self-loops only | No external molecular graph, KNN graph, or prior biological network imposed |
| Interpretability | Gradient SHAP | Post hoc feature attribution; candidate markers only |
| Software | PyTorch 2.4.1; Python analysis scripts | Main code in BC_LDS_2class/modules |
| Fold 1 test indices | 9 10 11 16 19 29 32 34 35 40 44 46 50 57 63 65 66 69 71 108 112 116 122 123 127 128 133 134 138 143 | Fold_1_predictions.json |
| Fold 2 test indices | 0 12 18 20 23 31 38 43 45 49 52 56 59 61 64 68 72 75 85 89 91 100 107 109 114 117 120 124 144 | Fold_2_predictions.json |
| Fold 3 test indices | 3 4 5 8 22 24 27 30 41 42 51 62 77 79 80 82 84 86 90 92 94 95 98 102 113 115 119 135 142 | Fold_3_predictions.json |
| Fold 4 test indices | 1 2 15 17 25 28 37 39 47 48 54 55 60 70 73 78 81 93 96 99 103 105 106 118 129 131 132 136 137 | Fold_4_predictions.json |
| Fold 5 test indices | 6 7 13 14 21 26 33 36 53 58 67 74 76 83 87 88 97 101 104 110 111 121 125 126 130 139 140 141 145 | Fold_5_predictions.json |

BC: breast cancer; LDS: Liver depression syndrome; SHAP: SHapley Additive exPlanations; KNN graph: k‑nearest‑neighbor.

**160** patients were screened

**13** Excluded

**4** Failed to meet the inclusion and exclusion criteria

**9** Refused to participate in this study

Breast cancer with Liver depression syndrome

(*n* = 41)

Breast cancer without Liver depression syndrome

(*n* = 31)

Non-breast cancer with Liver depression syndrome

(*n* = 40)

Non-breast cancer without Liver depression syndrome

(*n* = 35)

One of the samples failed the quality inspection

(*n* = 34)

**147** patients were enrolled

Figure S1 Flow diagram of participant selection.

Table S3 Clinical characteristics of the participants.

| **Characteristics** | **BC-LDS (*n* = 41)** | **BC-NLDS (*n* = 31)** | **NBC-LDS (*n* = 40)** | **NBC-NLDS (*n* = 34)** |
| --- | --- | --- | --- | --- |
| **Age (years)** | 50.00  (40.50, 56.00) | 54.00  (46.00, 58.00) | 35.00  (28.00, 48.75) | 39.50  (28.00, 47.25) |
| **Height (cm)** | 157.00  (152.50, 161.50) | 160.00  (155.00, 162.00) | 158.00  (155.25, 162.00) | 158.50  (156.00, 162.00) |
| **Weight (kg)** | 55.00  (50.25, 63.25) | 60.00  (53.50, 64.00) | 52.55  (49.00, 58.45) | 55.45  (49.30, 63.55) |
| **Marriage status (*n*, %)** |  |  |  |  |
| Single | 3 (7.32%) | 2 (6.45%) | 14 (35.00%) | 14 (41.18%) |
| Married | 37 (90.24%) | 29 (93.55%) | 26 (65.00%) | 19 (55.88%) |
| Widowed | 1 (2.44%) | 0 (0%) | 0 (0%) | 1 (2.94%) |
| **Menopausal status (*n*, %)** |  |  |  |  |
| Pre-menopause | 22 (53.66%) | 14 (45.16%) | 32 (80.00%) | 31 (91.18%) |
| Post-menopause | 19 (46.34%) | 17 (54.84%) | 8 (20.00%) | 3 (8.82%) |

Data are presented as the medians (p25, p75) or percentages (numbers of patients).

BC: breast cancer; NBC: non-breast cancer; LDS: Liver depression syndrome; NLDS: non-Liver depression syndrome.

Table S4 Clinical characteristics of the participants with breast cancer.

| **Characteristics** | **BC-LDS (*n* = 41)** | **BC-NLDS (*n* = 31)** |
| --- | --- | --- |
| **Tumor stage** |  |  |
| Stage 0 | 3 (7.32%) | 1 (3.23%) |
| Stage I | 3 (7.32%) | 7 (22.58%) |
| Stage II | 18 (43.90%) | 12 (38.71%) |
| Stage III | 11 (26.83%) | 8 (25.81%) |
| Stage IV | 6 (14.63%) | 2 (6.45%) |
| Unknown | 0 (0%) | 1 (3.23%) |
| **T staging** |  |  |
| Tis | 4 (9.76%) | 1 (3.23%) |
| T1 | 5 (12.20%) | 8 (25.81%) |
| T2 | 20 (48.78%) | 14 (45.16%) |
| T3 | 5 (12.20%) | 3 (9.68%) |
| T4 | 7 (17.07%) | 4 (12.90%) |
| Unknown | 0 (0%) | 1 (3.23%) |
| **N staging** |  |  |
| N0 | 13 (31.71%) | 14 (45.16%) |
| N1 | 16 (39.02%) | 11 (35.48%) |
| N2 | 1 (2.44%) | 0 (0%) |
| N3 | 10 (24.39%) | 5 (16.13%) |
| Unknown | 1 (2.44%) | 1 (3.23%) |
| **Histological grade** |  |  |
| Grade 1 | 0 (0%) | 1 (3.23%) |
| Grade 2 | 17 (41.46%) | 14 (45.16%) |
| Grade 3 | 20 (48.78%) | 13 (41.94%) |
| Unknown | 4 (9.76%) | 3 (9.68%) |
| **ER receptor status** |  |  |
| Positive | 31 (75.61%) | 22 (70.97%) |
| Negative | 10 (24.39%) | 8 (25.81%) |
| Unknown | 0 (0%) | 1 (3.23%) |
| **PR receptor status** |  |  |
| Positive | 31 (75.61%) | 17 (54.84%) |
| Negative | 9 (21.95%) | 12 (38.71%) |
| Unknown | 1 (2.44%) | 2 (6.45%) |
| **HER2 status** |  |  |
| Positive | 11 (26.83%) | 8 (25.81%) |
| Negative | 27 (65.85%) | 21 (67.74%) |
| Unknown | 3 (7.32%) | 2 (6.45%) |
| **Ki-67** |  |  |
| 0-20% | 6 (14.63%) | 9 (29.03%) |
| ＞20% | 33 (80.49%) | 20 (64.52%) |
| Unknown | 2 (4.88%) | 2 (6.45%) |

Data are presented as numbers of patients (percentages).

BC: breast cancer; LDS: Liver depression syndrome; NLDS: non-Liver depression syndrome.

Figure S2 The omics analysis of transcriptomic, proteomic, and metabolomic features in Liver depression syndrome in breast cancer.

(A) Number of differentially expressed genes (DEGs) identified from the transcriptome of four groups; annotation results of BC-LDS characteristic genes in the Gene Ontology (GO) and Kyoto Encyclopedia of Genes and Genomes (KEGG) databases. (B) Partial Least Squares Discriminant Analysis (PLS-DA) of 146 proteomics samples; number of differentially expressed proteins (DEPs) identified by proteomics; annotation of BC-LDS characteristic proteins in the GO and KEGG databases. (C) Principal Component Analysis (PCA) of 146 metabolomics samples (including QC samples); annotation of BC-LDS characteristic metabolites; number of differentially abundant metabolites (DAMs) identified by metabolomics; correlation heatmap among BC-LDS characteristic metabolites; KEGG annotation and topological analysis of BC-LDS characteristic metabolites. BC: breast cancer; LDS: Liver depression syndrome.

Table S5 Pathways associated with Liver depression syndrome of breast cancer identified through multi-omics analysis.

| **Pathways** | **Transcriptomic results** | **Proteomic results** | **Metabolomic results** |
| --- | --- | --- | --- |
| Steroid hormone biosynthesis | ENSG00000176387(HSD11B2) ENSG00000138759 (HSD3B2) ENSG00000124102 (CYP21A2) ENSG00000167971 (HSD17B3) ENSG00000173714 (AKR1C3) ENSG00000162068 (AKR1C1 ENSG00000169271 (CYP1B1) ENSG00000198416 (CYP2E1) ENSG00000134207 (NQO1) | ENSP00000216161.5 (HSD3B1) ENSP00000263897.4 (CYP21A2) ENSP00000232508.5 (HSD17B3) ENSP00000271663.7 (AKR1C3) ENSP00000284957.4 (AKR1C1) ENSP00000223127.3 (CYP1B1) | Tetrahydrocortisol Cholesterol Sulfate |
| Neuroactive ligand-receptor interaction | ENSG00000121853 (GHSR) ENSG00000121853 (HTR1D) ENSG00000123700 (HTR1F) ENSG00000162551 (GRIA4) ENSG00000169397 (CHRM3) ENSG00000169862 (OPRD1) ENSG00000176387 (GABRB3) ENSG00000176754 (GABRA5 ENSG00000177990 (GRIN2B） ENSG00000182676 (P2RX3) ENSG00000182752 (HRH3) ENSG00000185904 (AVPR1A)  ENSG00000197462 (TACR1) ENSG00000198416 (CHRNA4) | ENSP00000256969.2 (SPX) ENSP00000290765.4 (GSTT2) ENSP00000391121.2 (RDH13) ENSP00000261733.2 (ALDH2) | Noradrenaline（metab_7587） |
| cAMP signaling pathway | ENSG00000121853 (GHSR) ENSG00000121853 (CREB5) ENSG00000123700 (PDE4B) ENSG00000128322 (RAPGEF4,EPAC2) ENSG00000145777 (PDE7A) ENSG00000147647 (ADCY7) ENSG00000162551 (GRIA4) ENSG00000163633 (RAPGEF3/EPAC1) ENSG00000167971 (PDE4D) ENSG00000169397 (PRKACA) | ENSP00000244289.3 (LIPE, HSL) ENSP00000444467.1 (RAP1B) | Noradrenaline (metab_7587) |
| Tryptophan metabolism | ENSG00000169271 (IDO1) ENSG00000134207 (TDO2) ENSG00000124102 (KYNU) ENSG00000173714 (AADAT) ENSG00000116703 (KMO) ENSG00000138759 (CCBL1) ENSG00000198416 (TPH1) ENSG00000197915 (TPH2) ENSG00000142677 (DDC) ENSG00000124216 (MAOA) ENSG00000162068 (AOC1) ENSG00000106714 (GOT2) ENSG00000164047 (IL4I1) | ENSP00000261733.2 (ALDH2) | Indole-3-Acetic Acid (metab_1587) 2-Formamidobenzoic Acid (metab_8535) 3-(3-Indolyl)-2-Oxopropanoic Acid (metab_12941) |
| Chemical carcinogenesis-ROS | ENSG00000106714 (GSTK1) ENSG00000124102 (GSTP1) ENSG00000142677 (CYP1A1) ENSG00000169271 (CYP1B1) ENSG00000198416 (CYP2E1) ENSG00000134207 (NQO1) ENSG00000138759 (EPHX1) ENSG00000162068 (AKR1C1) ENSG00000173714 (AKR1C3) ENSG00000116703 (NOX4) ENSG00000124216 (SOD2) ENSG00000164047 (IL4I1) ENSG00000197462 (MGST1) ENSG00000197915 (GSTA4) ENSG00000104804 (PRDX1) ENSG00000105048 (PRDX6) ENSG00000100302 (TXNRD1) ENSG00000121853 (CAT) | ENSP00000290765.4 (GSTT2) ENSP00000301457.1 (NDUFA7) | Benzene Oxide (metab_13638) |
| Choline metabolism in cancer | ENSG00000104918 (CHKA) ENSG00000162068 (PCYT1A) ENSG00000116703 (PLA2G4A) ENSG00000123700 (PLD1) ENSG00000145777 (SLC44A1) | ENSP00000216061.5 (PLA2G4A) | Gpcho (20:5/22:4) (metab_2690) |

BC: breast cancer; LDS: Liver depression syndrome.

Table S6 Leakage-free repeated nested cross-validation performance from original full omics matrices.

| **Metric** | **Observed** | **Cluster bootstrap mean** | **95% CI lower** | **95% CI upper** |
| --- | --- | --- | --- | --- |
| AUC | 0.968 | 0.968 | 0.940 | 0.992 |
| PR-AUC | 0.949 | 0.950 | 0.900 | 0.987 |
| Accuracy | 0.966 | 0.966 | 0.943 | 0.984 |
| Sensitivity (BC-LDS) | 0.927 | 0.928 | 0.859 | 0.984 |
| Specificity (others) | 0.981 | 0.981 | 0.965 | 0.994 |
| F1 (BC-LDS) | 0.938 | 0.938 | 0.894 | 0.973 |
| Brier score | 0.037 | 0.037 | 0.021 | 0.055 |
| ECE_10_bins | 0.031 | 0.035 | 0.021 | 0.053 |

Abbreviations: AUC, area under the receiver operating characteristic curve; PR-AUC, area under the precision-recall curve; ECE, expected calibration error.

Table S7 Raw-matrix permutation test with complete fold-internal feature selection.

| **Metric** | **Observed** | **Null mean** | **Null SD** | **Null 95th percentile** | **Empirical p** |
| --- | --- | --- | --- | --- | --- |
| AUC | 0.975 | 0.503 | 0.045 | 0.591 | 0.0099 |
| PR-AUC | 0.975 | 0.304 | 0.044 | 0.379 | 0.0099 |
| Accuracy | 0.952 | 0.657 | 0.030 | 0.699 | 0.0099 |
| Sensitivity (BC-LDS) | 0.829 | 0.146 | 0.083 | 0.294 | 0.0099 |
| Specificity (others_ | 1.000 | 0.856 | 0.051 | 0.933 | 0.0099 |
| F1 (BC-LDS) | 0.907 | 0.185 | 0.086 | 0.329 | 0.0099 |

**Interpretation.** The leakage-free repeated nested cross-validation retained high internal discrimination, and the raw-matrix permutation test showed that the observed performance exceeded the corresponding random-label distributions. These findings support the presence of a nonrandom composite BC-LDS-associated molecular pattern. Nevertheless, because all analyses remain internally validated within the same prospective cohort, the model should be interpreted as an exploratory tool for candidate biomarker prioritization rather than as an externally validated diagnostic model.

Table S8 Stable SHAP-supported candidate features identified by bootstrap analysis (top-30 inclusion frequency ≥0.80).

| **Features** | **Omics** | **SHAP mean** | **Top30 bootstrap** | **Mean rank** |
| --- | --- | --- | --- | --- |
| CLPP | proteomic | 0.004388828 | 1 | 1.379 |
| RALGAPB | proteomic | 0.004264727 | 1 | 1.71 |
| CCZ1B | proteomic | 0.003645893 | 1 | 3.435 |
| LGALS8 | proteomic | 0.003502311 | 1 | 4.055 |
| EPB41L3 | proteomic | 0.002902274 | 1 | 8.115 |
| CEP131 | proteomic | 0.002844375 | 1 | 9.895 |
| ALG2 | proteomic | 0.002655164 | 1 | 13.666 |
| Sm(D17:1/16:0) | metabolomic | 0.002575789 | 1 | 1.114 |
| Phosalone | metabolomic | 0.002284949 | 1 | 2.694 |
| 2-(2-Butoxyethoxy)Ethyl Acetate | metabolomic | 0.002168429 | 1 | 4.291 |
| Farnesyl Acetone | metabolomic | 0.002123989 | 1 | 5.219 |
| Lpa(20:4) | metabolomic | 0.002050637 | 1 | 6.099 |
| N,N-Bis(2-Chloroethyl)-N',N''-Diphenylphosphoric Triamide | metabolomic | 0.002016676 | 1 | 7.275 |
| Platelet-Activating Factor | metabolomic | 0.001982808 | 1 | 7.894 |
| Sm(14:0_2O/26:2) | metabolomic | 0.001867986 | 1 | 11.639 |
| ENSG00000165733 | transcriptome | 0.001495326 | 1 | 1.5 |
| ENSG00000159202 | transcriptome | 0.001452377 | 1 | 1.82 |
| ENSG00000122557 | transcriptome | 0.001346564 | 1 | 3.509 |
| ENSG00000170515 | transcriptome | 0.001268657 | 1 | 5.141 |
| ENSG00000111237 | transcriptome | 0.001221679 | 1 | 6.481 |
| ENSG00000168661 | transcriptome | 0.001219064 | 1 | 6.907 |
| CD40LG | proteomic | 0.002616847 | 0.999 | 14.62062062062062 |
| Luteolin 3'-Glucuronide | metabolomic | 0.00197262 | 0.999 | 8.622622622622623 |
| MLX | proteomic | 0.003115231 | 0.998 | 6.557114228456914 |
| Petroselinic Acid | metabolomic | 0.001912647 | 0.998 | 10.643286573146293 |
| ENSG00000179222 | transcriptome | 0.001140737 | 0.998 | 9.856713426853707 |
| 8-Oxohexadecanoic Acid | metabolomic | 0.001935803 | 0.997 | 9.688064192577734 |
| ENSG00000106069 | transcriptome | 0.001152867 | 0.997 | 10.060180541624874 |
| GSTT2 | proteomic | 0.00276744 | 0.996 | 11.02710843373494 |
| Naldemedine | metabolomic | 0.001817911 | 0.996 | 13.88855421686747 |
| ENSG00000143499 | transcriptome | 0.001232081 | 0.996 | 6.920682730923695 |
| ENSG00000149480 | transcriptome | 0.00113103 | 0.995 | 10.266331658291458 |
| 4-Chlorobenzoic Acid | metabolomic | 0.001816851 | 0.991 | 14.026236125126136 |
| ENSG00000136875 | transcriptome | 0.001121726 | 0.989 | 11.19211324570273 |
| ENSG00000138801 | transcriptome | 0.001076691 | 0.987 | 14.126646403242148 |
| PTGES2 | proteomic | 0.002643167 | 0.985 | 14.32994923857868 |
| CXorf38 | proteomic | 0.002563718 | 0.984 | 16.410569105691057 |
| PYM1 | proteomic | 0.002857721 | 0.983 | 10.387589013224822 |
| ENSG00000142444 | transcriptome | 0.001039018 | 0.981 | 16.4841997961264 |
| ENSG00000101452 | transcriptome | 0.001045743 | 0.98 | 16.14387755102041 |
| RAE1 | proteomic | 0.002607764 | 0.971 | 14.790937178166839 |
| PPP6R2 | proteomic | 0.002499073 | 0.965 | 18.784455958549223 |
| RAP1B | proteomic | 0.002693136 | 0.957 | 13.307210031347962 |
| GPCPD1 | proteomic | 0.002573095 | 0.955 | 16.186387434554973 |
| RABGEF1 | proteomic | 0.002451371 | 0.955 | 19.981151832460732 |
| LIMD1 | proteomic | 0.002518762 | 0.954 | 17.349056603773583 |
| ENSG00000175470 | transcriptome | 0.001023797 | 0.952 | 17.6281512605042 |
| 3-Ethyl-4-(9-Hydroxy-4,6,8,10-Tetramethyl-7-Oxododec-4-En-2-Yl)Oxetan-2-One | metabolomic | 0.001811054 | 0.949 | 13.873551106427819 |
| TXNL1 | proteomic | 0.002544875 | 0.947 | 17.43400211193242 |
| ENSG00000118705 | transcriptome | 0.001029912 | 0.946 | 17.192389006342495 |
| 46-Dimethylcurvulinic Acid | metabolomic | 0.001699734 | 0.945 | 19.125925925925927 |
| 2,5-Dihydroxybenzenesulfonic Acid | metabolomic | 0.001756349 | 0.936 | 16.23611111111111 |
| WASHC1 | proteomic | 0.002462709 | 0.933 | 20.027867095391212 |
| Sm(8:1_2O/34:2) | metabolomic | 0.00171531 | 0.931 | 18.50375939849624 |
| ENSG00000129480 | transcriptome | 0.001029441 | 0.927 | 16.644012944983817 |
| ENSG00000107485 | transcriptome | 0.001003001 | 0.922 | 19.746203904555315 |
| 2-Palmitoylglycerol | metabolomic | 0.001700136 | 0.904 | 18.825221238938052 |
| CFAP20 | proteomic | 0.002447305 | 0.901 | 19.457269700332965 |
| (Z)-3-Hydroxydodec-5-Enoic Acid | metabolomic | 0.001706613 | 0.887 | 18.42164599774521 |
| SIGIRR | proteomic | 0.002454248 | 0.883 | 19.612684031710078 |
| ENSG00000140718 | transcriptome | 0.000994401 | 0.869 | 20.53970080552359 |
| ENSG00000272606 | transcriptome | 0.001006381 | 0.862 | 19.03828306264501 |
| RAB23 | proteomic | 0.002428417 | 0.847 | 20.1357733175915 |
| ENSG00000008405 | transcriptome | 0.000986621 | 0.84 | 20.635714285714286 |
| Gamma-Glutamyltyrosine | metabolomic | 0.001709158 | 0.83 | 17.35903614457831 |

SHAP: SHapley Additive exPlanations.

Figure S3 Model performance derived from sensitivity analysis.

AUC: area under curve.

Table S9 Summary of differential multi‑omics features identified from factorial‑based statistical analyses.

| Analysis | Omics | Features tested | *p* < 0.05 | FDR < 0.10 | FDR < 0.05 | Top features | Top *p* value | FDR |
| --- | --- | --- | --- | --- | --- | --- | --- | --- |
| BC-LDS vs BC-NLDS | metabolome | 1415 | 100 | 0 | 0 | Sm(14:0_2O/26:2) | 0.0008674379423526478 | 0.5611852025518149 |
| BC-LDS vs BC-NLDS | protein | 5044 | 846 | 500 | 431 | SLC27A3 | 2.9578406667142216e-23 | 1.4919348322906532e-19 |
| BC-LDS vs BC-NLDS | transcriptome | 36446 | 1925 | 0 | 0 | ENSG00000271392 | 5.696122350057121e-06 | 0.20760087517018183 |
| LDS adjusted for cancer | metabolome | 1415 | 46 | 0 | 0 | 3,4-Dimethyl-5-Pentyl-2-Furanpropanoic Acid | 0.0024901675537197165 | 0.9036084075895271 |
| LDS adjusted for cancer | protein | 5044 | 813 | 521 | 477 | PAPSS1 | 2.494169776776823e-44 | 1.2580592354062295e-40 |
| LDS adjusted for cancer | transcriptome | 36446 | 2714 | 0 | 0 | ENSG00000179627 | 7.116787335087948e-05 | 0.66061637610587 |
| Cancer by LDS interaction | metabolome | 1415 | 67 | 0 | 0 | 3-(Cyclohexylamino)-2-Hydroxy-1-Propanesulfonic Acid | 0.0001754992370690946 | 0.2045518006497605 |
| Cancer by LDS interaction | protein | 5044 | 1000 | 695 | 569 | MLX | 6.354058278360965e-42 | 3.204986995605271e-38 |
| Cancer by LDS interaction | transcriptome | 36446 | 1352 | 0 | 0 | ENSG00000238251 | 0.00011663819646120024 | 0.998843180841664 |

BC: breast cancer; LDS: Liver depression syndrome; FDR: false discovery rate.

Table S10 Robustness analyses of group differences for four target biomarkers.

| **Biomarker** | **Group comparison** | **Raw analysis (P)** | **IQR outliers removed** | **IQR analysis (P)** | **Bootstrap 95% CI** |
| --- | --- | --- | --- | --- | --- |
| TRIM56 | BC-LDS vs NBC-NLDS | <0.001 | 2/7 | <0.001 | −107.450 to −39.685 |
|  | BC-LDS vs BC-NLDS | <0.001 | 2/1 | <0.001 | −263.469 to −122.983 |
|  | NBC-LDS vs NBC-NLDS | 0.0014 | 3/7 | <0.001 | 104.992 to 282.652 |
| AXL | BC-LDS vs NBC-NLDS | 0.006 | 1/1 | 0.005 | −1106.270 to −167.445 |
|  | BC-LDS vs BC-NLDS | 0.001 | 1/2 | <0.001 | 65.821 to 806.854 |
|  | NBC-LDS vs NBC-NLDS | 0.003 | 3/1 | <0.001 | −1348.067 to −526.730 |
| SIGIRR | BC-LDS vs NBC-NLDS | 0.877 | 3/6 | 0.483 | −0.345 to 0.440 |
|  | BC-LDS vs BC-NLDS | 0.023 | 3/3 | 0.007 | −0.000 to 0.697 |
|  | NBC-LDS vs NBC-NLDS | 0.008 | 9/6 | <0.001 | −0.750 to −0.078 |
| STING | BC-LDS vs NBC-NLDS | 0.008 | 5/7 | 0.013 | −2.300 to −0.095 |
|  | BC-LDS vs BC-NLDS | 0.345 | 5/3 | 0.194 | −0.156 to 0.137 |
|  | NBC-LDS vs NBC-NLDS | 0.540 | 7/7 | 0.593 | −1.963 to 0.336 |

IQR: Interquartile range; CI: confidence interval.
